# Effects of Bang® Keto Coffee Energy Drink on Metabolism and Exercise Performance in Resistance-Trained Adults: A Randomized, Double-blind, Placebo-controlled, Crossover Study

**DOI:** 10.1186/s12970-020-00374-5

**Published:** 2020-08-24

**Authors:** Patrick S. Harty, Matthew T. Stratton, Guillermo Escalante, Christian Rodriguez, Jacob R. Dellinger, Abegale D. Williams, Sarah J. White, Robert W. Smith, Baylor A. Johnson, Mark B. Sanders, Grant M. Tinsley

**Affiliations:** 1 Energy Balance & Body Composition Laboratory, Department of Kinesiology & Sport Management, Texas Tech University, Lubbock, TX 79424 USA; 2 California State University, San Bernardino, California USA

**Keywords:** Energy drinks, Metabolism, Caffeine, Protein, Performance

## Abstract

**Background:**

Energy drinks are often consumed by the general population, as well as by active individuals seeking to enhance exercise performance and augment training adaptations. However, limited information is available regarding the efficacy of these products. Thus, the purpose of this study was to determine the effects of a commercially available caffeine- and protein-containing energy drink on metabolism and muscular performance.

**Methods:**

Sixteen resistance-trained males (*n* = 8; mean ± SD; age: 22.4 ± 4.9 years; body mass: 78.8 ± 14.0 kg; body fat: 15.3 ± 6.4%) and females (*n* = 8; age: 24.5 ± 4.8 years; body mass: 67.5 ± 11.9 kg; body fat: 26.6 ± 7.1%) participated in this randomized, double-blind, placebo-controlled, crossover study. Following a familiarization visit, participants completed two identical visits to the laboratory separated by 5–10 days, each of which consisted of indirect calorimetry energy expenditure (EE) assessments before and after consumption of the beverage (Bang® Keto Coffee; 130 kcal, 300 mg caffeine, 20 g protein) or placebo (30 kcal, 11 mg caffeine, 1 g protein) as well as after exercise testing. In addition, participants’ subjective feelings of energy, fatigue, and focus as well as muscular performance (leg press one-repetition maximum and repetitions to fatigue, maximal isometric and isokinetic squat testing) were assessed. Multiple repeated measures ANOVAs with Tukey *post-hoc* tests were used to analyze data. Estimates of effect size were quantified via partial eta squared (η_P_^2^) and Hedge’s g.

**Results:**

A significant interaction effect was identified for EE (*p* < 0.001, η_P_^2^ = 0.52) but not respiratory exchange ratio (*p* = 0.17, η_P_^2^ = 0.11). Following consumption of the beverage, EE was 0.77 kcal·min^− 1^ greater than placebo at the post-beverage time point (*p* < 0.001) and 0.37 kcal·min^− 1^ greater than placebo at the post-exercise time point (*p* = 0.011). However, no between-condition differences were detected for any subjective or muscular performance outcomes.

**Conclusions:**

The results of this study suggest that consumption of the energy drink had minimal effects on lower-body muscular performance and subjective factors in the context of a laboratory setting. However, the beverage was found to significantly increase energy expenditure compared to placebo immediately following ingestion as well as during the recovery period after an exercise bout, suggesting that active individuals may improve acute metabolic outcomes via consumption of a caffeine- and protein-containing energy drink.

**Trial registration:**

This trial was prospectively registered at ClinicalTrials.gov (Identifier: NCT04180787; Registered 29 November 2019).

## Introduction

Energy drinks and related caffeine-containing energy products are often consumed by the general population, adolescents, and individuals seeking to enhance exercise performance, augment training adaptations, and assist with weight loss [1–3]. These products typically contain caffeine and a blend of additional ingredients such as taurine, guarana, amino acids, and B-vitamins which are purported to improve energy levels, metabolism, and exercise performance [4]. However, it is important to note that the majority of the acute performance and metabolic benefits of energy drinks appear to be mediated by their caffeine and/or carbohydrate content [4]. To date, several investigations have assessed the efficacy of energy drinks and related products to improve muscular strength and power [5, 6], muscular endurance [7], behavioral alertness [8], and sport-specific performance [9], with generally-positive results [10]. In some cases, researchers are faced with significant challenges when attempting to quantify the ergogenic potential of a given energy product, as energy drink labels will often list many ingredients in “proprietary blends,” with exact amounts not provided [4]. Even when proprietary blends are not employed, the wide diversity of products on the market indicates that targeted research should be conducted to determine the actual efficacy of commercially available formulations.

A variety of investigations have demonstrated that the consumption of caffeine-containing energy drinks may acutely increase metabolic rate [11–13], which could potentially result in preferential changes in body composition over time with prolonged use [14]. Similar short-term metabolic effects have also been noted following the acute consumption of supplemental protein prior to an exercise session [15–17]. For example, Wingfield and colleagues [16] examined the metabolic impact of supplemental protein ingestion prior to aerobic exercise, high-intensity interval training, and resistance training. Compared to a calorie-matched carbohydrate condition, protein ingestion resulted in greater energy expenditure and a lower respiratory exchange ratio, suggesting increased fat oxidation and/or decreased exogenous carbohydrate oxidation, during the hour following exercise. Similarly, Gieske et al. [15] demonstrated that participants who consumed whey protein or casein protein prior to 30 min of moderate-intensity treadmill exercise had significantly greater within-group changes in energy expenditure following exercise compared to those who consumed a calorie-matched carbohydrate solution or a non-caloric control. Taken together, these results suggest that caffeine-containing products may provide beneficial metabolic effects, particularly when consumed in conjunction with other dietary agents such as additional protein.

Currently, more information is needed to determine the combined effect of acute protein intake and caffeinated energy drink consumption prior to an exercise bout on metabolic outcomes before and after the bout. Similarly, further information is required to determine the effectiveness of a caffeine and protein-containing energy drink on isometric, isokinetic, and isotonic measures of muscular performance. Many individuals also consume energy drinks to increase feelings of energy and reduce subjective fatigue, although there are limitations to estimating the real-world impact of energy drinks on these outcomes in the laboratory setting. Importantly, as females have traditionally been underrepresented in sports science research [18], it is also critical to examine the aforementioned outcomes in both males and females. Thus, the goal of the present study was to quantify the acute ergogenic and metabolic effects of a commercially available caffeine and protein-containing energy drink in resistance-trained males and females. A secondary purpose of this investigation was to quantify changes in participant ratings of perceived energy, fatigue, and focus following consumption of the beverages. It was hypothesized that acute ingestion of the energy drink would improve muscular performance and result in significantly elevated measures of resting metabolic rate.

## Methods

### Overview

**Fig. 1:**
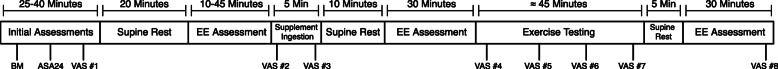
Testing Session Overview. ASA24: Self-administered dietary recall; BM: Body mass assessment; EE Assessment: Energy expenditure assessment; VAS: Visual analog scale

This randomized, double-blind, placebo-controlled, crossover study consisted of three visits to the laboratory. During an initial screening and familiarization visit, participants were informed of the study requirements, completed a written informed consent document, and underwent preliminary assessment of height, weight, and body composition via bioelectrical impedance analysis. In addition, each participant was familiarized with the performance tests that would be completed throughout the study. After completion of the screening visit, all participants underwent two morning testing sessions, each separated by 5–10 days. During each testing visit, participants reported to the laboratory after an overnight (≥ eight-hour) fast from all calorie-containing foods and fluids and an eight-hour abstention from caffeine, nicotine, and alcohol. In addition, participants were instructed to abstain from intense lower-body resistance exercise for at least 48 h prior to each testing session, and to completely abstain from all exercise for at least 24 h prior to each session. Upon arrival, participants underwent body weight assessment and completed a 24-h dietary recall to allow for replication of dietary intake on the day prior to the session. Following completion of the dietary recall, participants completed three visual analog scales (VAS) rating their feelings of energy, fatigue, and focus and then underwent a baseline resting metabolic rate (RMR) assessment via indirect calorimetry. Next, participants consumed either the energy drink or placebo in a randomized and double-blind fashion. Additional VAS measurements were collected before and after ingestion. After 10 min of supine rest, participants underwent a second RMR assessment and then completed a fourth VAS questionnaire. Following the questionnaire, participants performed a five-minute standardized warmup consisting of bodyweight squats, lunges, and low-to-moderate effort squat jumps. Next, participants performed three maximal isometric squats at 150 and 120 degrees of knee extension using a mechanized squat device (Exerbotics eSq, Tulsa, OK). Following the isometric testing, participants performed a three-repetition, maximal isokinetic squat protocol to assess maximal eccentric and concentric force production, followed by a fifth VAS questionnaire. Participants then performed one-repetition maximum (1RM) testing and repetitions to fatigue on an angled leg press, using a load equivalent to 2x body mass for males, and 1.5x body mass for females. After completing the repetitions to fatigue, the participants then repeated the isometric and isokinetic squat testing protocols, followed by another VAS questionnaire. Immediately thereafter, participants were positioned to undergo a final RMR assessment, which commenced 5 min after completion of the VAS questionnaire. Following the final RMR assessment, a final VAS questionnaire was completed. An overview of data collection procedures is provided in Fig. 1. Five to ten days after completing the first testing session, participants reported to the lab and completed an identical testing protocol with the alternate beverage condition. This study was approved by the Texas Tech University Institutional Review Board (IRB00000276), and all participants signed the approved consent document prior to participation. This trial was prospectively registered on clinicaltrials.gov (protocol ID: NCT04180787; registered 29 November 2019).

### Participants

Sixteen resistance-trained adults (8 M, 8F; Table 1) were recruited to participate in this study. All participants were required to be between the ages of 18–40, generally healthy (defined as an absence of any disease or medical condition which could potentially be negatively affected by consumption of the product), and regular caffeine consumers (defined as consumption of at least 200 mg·day^− 1^). In addition, all participants were required to be resistance-trained, defined as completing three or more resistance training sessions per week for at least 1 year and including at least weekly lower body training through a multi-joint exercise such as the squat or leg press. Exclusion criteria included taking any prescription medication which would make participation unsafe or influence study outcomes, an inability to complete lower-body resistance exercise due to injury or medical condition, allergy to any of the ingredients in the energy drink or placebo, self-reported caffeine sensitivity characterized by unwanted side effects, pregnancy or active breastfeeding, and self-reported claustrophobia.

**Table 1:** Participant Characteristics

		Mean ± SD
Age (years)	*Males (n = 8)*	22.4 ± 4.9
	*Females (n = 8)*	24.5 ± 4.8
	**Total (*N*** **= 16)**	**23.4 ± 4.8**
Height (cm)	*Males (n = 8)*	175.2 ± 7.9
	*Females (n = 8)*	166.2 ± 6.2
	**Total (*N*** **= 16)**	**170.7 ± 8.3**
Body Mass (kg)	*Males (n = 8)*	78.8 ± 14.0
	*Females (n = 8)*	67.5 ± 11.9
	**Total (*N*** **= 16)**	**73.1 ± 13.8**
Body Fat (%)	*Males (n = 8)*	15.3 ± 6.4
	*Females (n = 8)*	26.6 ± 7.1
	**Total (*N*** **= 16)**	**21.0 ± 8.8**

### Procedures

#### Body composition assessment

Body composition was assessed during the initial screening visit via bioelectrical impedance analysis using a freestanding, eight-point multifrequency bioelectrical impedance device (Inbody 770, Inbody USA, Cerritos, CA). Participants removed their shoes and socks and wore light athletic clothing while undergoing the assessment.

#### Energy expenditure assessment

Energy expenditure (EE) and respiratory exchange ratio (RER) were assessed via indirect calorimetry (ParvoMedics TrueOne 2400, ParvoMedics, Sandy, UT). Prior to each research visit, daily gas flow and gas analyzer calibrations were performed per manufacturer guidelines. During each resting metabolic rate assessment, participants reclined in a supine position with a clear plastic hood and drape placed over their shoulders and were instructed to remain motionless but awake throughout the duration of the test. If desired, a blanket was provided to participants during the test. All assessments were conducted in the same climate-controlled laboratory with the lights dimmed. Prior to the initial EE assessment, participants rested for 20 min followed by at least 10 min of expired gas collection, with the first 5 min of the test discarded according to the recommendations of Fullmer and colleagues [19]. The initial resting EE assessment was continued until the coefficient of variation (CV) for EE values was less than 10% and the CVs for measured VO2 and VCO2 were less than 5% for a period of five consecutive minutes. The five-minute period which met these criteria was recorded. During the pre-exercise and post-exercise metabolic assessments, expired gases were collected continuously for 30 min to better capture the acute variation in metabolic outcomes due to the thermic effect of food, acute thermogenic effects of caffeine, and excess post-exercise oxygen consumption. Following ingestion of the energy drink or placebo, participants rested in the supine position for 10 min prior to the pre-exercise EE assessment. After completing the final performance assessments, a period of up to 5 min of supine rest was also implemented prior to the post-exercise EE assessment. The observed CVs for EE in the three assessments were: 2.7 ± 0.9% (pre-beverage), 5.3 ± 2.6% (post-beverage), and 7.5 ± 2.5% (post-exercise). Due to the likelihood that monitoring EE assessments could potentially compromise the blinding of research personnel, a single researcher who was not directly involved in muscular performance testing monitored all EE assessments.

#### Isometric and isokinetic testing

Isometric and isokinetic performance tests were conducted using a mechanized squat device (Exerbotics eSq, Tulsa, OK). Prior to starting the testing battery, participants completed a five-minute warm up consisting of bodyweight air squats, alternating lunges, and jump squats. Upon completion of the warm up, participants removed their shoes and stood in a self-selected foot position on the squat platform, which was noted and replicated at all subsequent testing sessions. During the tests, participants stood in an upright position with the pads of the squat device resting on the upper trapezius. Maximal isometric force production tests were conducted at 150 and 120 degrees of knee extension. During each isometric test, participants performed two practice warmup attempts, one at approximately 50% of maximal effort and one at approximately 75% of maximal effort, followed by two maximal isometric squat attempts for which the participant was instructed to push as hard and as fast as possible. Following the isometric assessments, participants completed a three-repetition maximal isokinetic force production test, with a range of motion from 90 degrees to 150 degrees of knee extension. Each repetition consisted of a four-second eccentric phase followed by a four-second concentric phase, with brief pauses at the 90 degree and 150-degree positions. Participants were instructed to perform the first repetition at approximately 50% effort, and this repetition was not included in data analysis. The remaining two repetitions were performed with maximal effort, and participants were provided with strong verbal encouragement throughout all muscular performance assessments. Force data during the isometric and isokinetic tests was sampled from a load cell at 1 kHz (MP1150WSW, Biopac Systems Inc., Santa Barbara, CA) and later processed using a custom software program (LabVIEW Version 11.0, National Instruments, Austin, TX). All analyses were conducted using a scaled and filtered force signal (low-pass filtered with a 10-Hz cutoff, zero-phase lag, fourth-order Butterworth filter). To determine isometric peak force, the highest 500 ms epoch was identified and quantified. In addition, peak rate of force development (RFD_peak_), early rate of force development (RFD_50_; i.e., RFD in the first 50 ms), and late rate of force development (RFD_200_; i.e., RFD in the first 200 ms) were determined. The initiation of force production for RFD variables was determined using the automated method, with the onset specified as 1% of the maximal force produced. To quantify isokinetic peak force, the highest mean 25 ms epochs for concentric and eccentric portions of the repetition were identified. These values obtained from the second and third repetitions of the three-repetition test were averaged for analysis. The reliability of these procedures in our laboratory has previously been described [20].

#### Isotonic testing

Lower-body strength was also evaluated via angled leg press 1RM testing. Immediately after completion of the initial isometric and isokinetic testing protocols, participants performed a leg press warmup protocol according to the recommendations set forth by the National Strength and Conditioning Association [21]. In short, a set of eight-ten repetitions was performed at 40–60% perceived 1RM, followed by a set of three-five repetitions at 60–80% perceived 1RM, followed by a set of two-three repetitions with an additional 10–20% 1RM added. After completing the warmup sequence, participants performed up to five attempts, with 3 min of rest between attempts, until a 1RM was determined. After 3 min of rest following the final 1RM attempt, participants performed maximal leg press repetitions to fatigue using a load equivalent to 2x body mass for males, and 1.5x body mass for females. The load used for repetitions to fatigue was standardized during both testing sessions. Participants were allowed to perform repetitions at a self-selected cadence but were instructed not to pause between repetitions; the test was terminated if participants were unable to comply with this instruction. For all repetitions, participants were required to achieve a ~ 90-degree knee angle before initiating the concentric portion of the movement. Failure to meet this requirement resulted in a repetition being considered invalid. To promote further standardization, participants were instructed to wear the same footwear during each testing session, and their foot positions were recorded and replicated during all leg press attempts. All testing was performed on a plate-loaded angled leg press machine (EliteFTS™ Leg Press) under direct researcher supervision.

#### Subjective measures

Subjective ratings of energy, fatigue, and focus were collected at seven time points during each visit: upon arrival to the laboratory, immediately prior to beverage ingestion, immediately post-ingestion, immediately prior to isometric/isokinetic testing, immediately prior to leg press testing, immediately after leg press testing, immediately after the second bout of isometric/isokinetic testing, and immediately after the final metabolic assessment. During each time point, participants completed visual analog scales (VAS) administered via electronic application on a tablet (VasQ). All visual analog scales were grounded, with relevant descriptors on either extreme of the line but no additional markings on the line itself. All VAS scores were expressed in a score ranging from 0 to 100, with zero being the minimum score and 100 being the maximal score.

#### Beverage ingestion

During each testing session, participants ingested either a commercially available, caffeine- and protein-containing energy drink (Bang® Keto Coffee, Hazelnut flavor) or a flavor and volume-matched placebo in a randomized, placebo-controlled, counter-balanced, crossover fashion. Randomization of the beverage ingestion order was performed using the *randomizeBE* software package in R and was counterbalanced within each sex. Each beverage was mixed with ice and given to the participants in an opaque shaker bottle, and participants were instructed to drink their assigned beverage within 5 min without commenting on its flavor or texture for the duration of the visit. Both the energy drink and flavor-matched placebo were provided by the manufacturer in unmarked cans with the exception of “A” and “B” labels. The manufacturer’s laboratory producing the beverages also provided the nutritional facts of the energy drink and placebo beverages. The energy drink contained approximately 130 kcal, 2 g fat, 5 g carbohydrate, 20 g protein, and 300 mg caffeine and was identical to the commercially available version sold in stores, while the placebo contained approximately 30 kcal, 1 g fat, 7 g carbohydrate, 1 g protein, and 11 mg caffeine and contained decaffeinated flavoring used in the commercial product. The full ingredient list for the energy drink was: coffee, protein blend (milk protein concentrate, whey protein isolate), natural and artificial flavors, cellulose cell, Straight 8® (caprylic triglyceride), cellulose gum, mono and diglycerides, sodium tripolyphosphate, sweet cream, carrageenan, caffeine, BCAAs (L-leucine, L-isoleucine, L-valine), and sucralose. The placebo beverage contained the same flavoring agents along with decaffeinated coffee in order to match the taste of the energy drink.

#### Dietary recall

Participants were asked to follow the same self-selected dietary intake for the single day prior to each testing visit. Each participant completed the Automated 24-h Dietary Assessment Tool (ASA24®) [22] upon arrival to the lab in order to assist with recalling the previous day’s diet. ASA24® is a web-browser based tool provided by the National Cancer Institute that utilizes an automated multiple-pass method to collect detailed dietary information. A copy of the previous day’s dietary recall was provided to each participant after the first testing visit for replication prior to the second visit. For standardization purposes, ASA24® was performed at the beginning of both testing visits.

### Statistical analysis

Analysis of variance (ANOVA) with repeated measures was used to examine potential changes in outcome variables across time. The prevalence of missing data ranged from 0 to 6%, with the primary reason for missing data being technical failure of a device. Multiple imputation with 100 iterations was performed using the *mice* package [23] in order to estimate the missing values and preserve the full sample size for each outcome variable. ANOVA was performed using the *afex* package [24], and η_P_^2^ effect sizes were generated. Normality of residuals was assessed by visual examination of quantile-quantile plots. Data that were not normally distributed were transformed using the *BestNormalize* package [25]. In the event of sphericity violations, Greenhouse-Geisser corrections were employed. Follow up for significant effects was performed using pairwise comparisons with Tukey adjustment via the *emmeans* package [26]. Although ANOVA procedures and follow up pairwise comparisons were based on transformed data when appropriate due to normality violations, raw data are presented in the figures to aid interpretability. Leg press variables were analyzed using paired-samples t-tests. Statistical significance was accepted at *p* ≤ 0.05, although raw data and effect sizes are displayed to facilitate holistic interpretation of results. Data were analyzed using R (v. 3.6.1) [27]. All data analysis was conducted and finalized prior to unblinding of beverage conditions.

## Results

Following the initial screening process, 19 individuals were deemed eligible for participation, signed the informed consent document, and began the study. Three participants withdrew from the study (one due to onset of minor illness unrelated to the study, and two due to scheduling conflicts). Sixteen participants (8 males, 8 females) completed all aspects of data collection. Participant demographics are presented in Table 1.

### Indirect Calorimetry

#### Energy expenditure

**Fig. 2:**
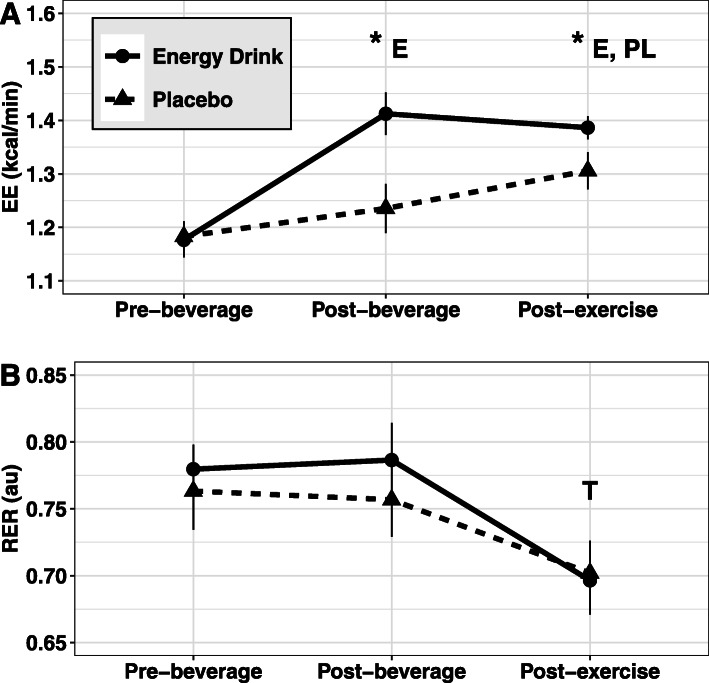
Metabolism. Changes in resting metabolic rate (RMR; panel **a**) and respiratory exchange ratio (RER; panel **b**), assessed via indirect calorimetry, are displayed. For RMR, a significant condition by time interaction was observed using ANOVA with repeated measures. Subsequently, Tukey post-hoc comparisons were performed. Asterisks indicate significant differences between conditions at the specified time point, while E and PL indicate significant differences in the energy drink or placebo conditions, respectively, relative to the pre-beverage value. For RER, a significant main effect for time was observed. T indicates a significant difference from the pre-beverage time point in both groups combined. Error bars indicate the 95% confidence intervals for within-subjects SE due to the repeated-measures design of this study [24, 28]

Main effects for condition (*p* < 0.001, η_P_^2^ = 0.68) and time (*p* < 0.001, η_P_^2^ = 0.81) were identified for EE, as well as a significant condition by time interaction (*p* < 0.001, η_P_^2^ = 0.52; Fig. 2a). In the placebo condition, EE did not increase significantly from pre-beverage to post-beverage (*p* = 0.24). However, EE increased by approximately 0.34 kcal·min^− 1^ from post-beverage to post-exercise (*p* = 0.025). Additionally, post-exercise EE was approximately 0.58 kcal·min^− 1^ higher than the baseline (pre-beverage) EE (*p* < 0.001).

Following consumption of the energy drink, EE increased approximately 1.10 kcal·min^− 1^ from pre-beverage to post-beverage (*p* < 0.001) and remained elevated by approximately 1.04 kcal·min^− 1^ at post-exercise (*p* < 0.001). However, there was no difference between post-beverage and post-exercise (*p* = 0.96). There were no baseline differences in EE between the energy drink or placebo conditions. EE following consumption of the energy drink was 0.77 kcal·min^− 1^ greater than placebo at the post-beverage time point (*p* < 0.001) and 0.37 kcal·min^− 1^ greater than placebo at the post-exercise time point (*p* = 0.011).

#### Respiratory exchange ratio

Significant main effects for time (*p* < 0.001, η_P_^2^ = 0.63) but not condition (*p* = 0.28, η_P_^2^ = 0.07) were identified for respiratory exchange ratio (Fig. 2b). A significant condition by time interaction was not identified for RER (*p* = 0.17, η_P_^2^ = 0.11). Tukey post-hoc comparisons showed that RER was significantly lower at the post-exercise time point compared to pre-beverage (*p* < 0.001) and post-beverage (*p* < 0.001) with both conditions combined.

### Muscular performance

#### Isokinetic testing

**Fig. 3:**
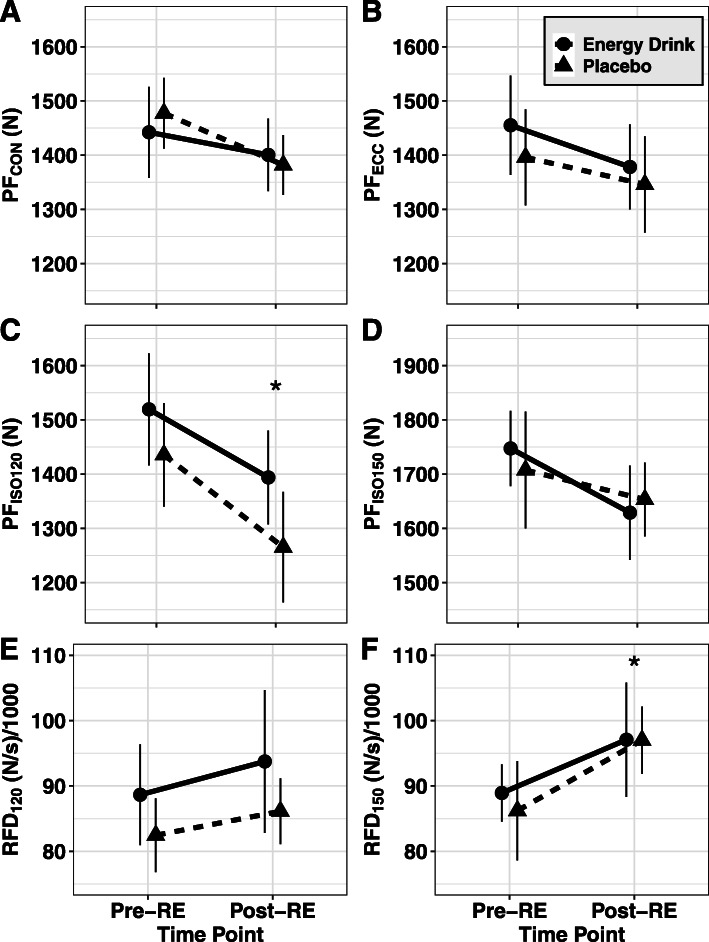
Squat Performance. Values for isokinetic and isometric performance on the mechanized squat device are displayed, with both time points occurring after beverage ingestion. Pre-RE tests were performed following the second indirect calorimetry assessment and prior to the resistance exercise (RE) testing, while post-RE tests occurred immediately after RE (leg press) testing. Values are displayed for peak concentric force (PF_CON_; panel **a**), peak eccentric force (PF_ECC_; panel **b**), peak isometric force at the 120-degree knee angle (PF_ISO120_; panel **c**), peak isometric force at the 150-degree knee angle (PF_ISO150_; panel **d**), peak rate of force development at the 120-degree knee angle (RFD_120_; panel **e**), and peak rate of force development at the 150-degree knee angle (RFD_150_; panel **f**). Data were analyzed using ANOVA with repeated measures, along with Tukey post-hoc comparisons. A main effect of time was present for PF_ISO120_ (*p* < 0.001, η_P_^2^ = 0.65), along with a trend for a main effect of condition (*p* = 0.07, η_P_^2^ = 0.19). Additionally, a main effect of time was present for RFD_150_ (*p* = 0.028, η_P_^2^ = 0.28). Error bars indicate the 95% confidence intervals for within-subjects SE due to the repeated-measures design of this study [24, 28]

A trend for a significant main effect of attempt (*p* = 0.07, η_P_^2^ = 0.20) but not condition (*p* = 0.88, η_P_^2^ = 0.00) was identified for concentric force production during isokinetic testing (Fig. 3a). However, a significant interaction effect was not identified (*p* = 0.20, η_P_^2^ = 0.11). Effect size analysis showed a negligible effect of condition on either concentric attempt (g = 0.04 and 0.05, respectively). No significant main (*p* > 0.07) or interaction (*p* = 0.71, η_P_^2^ = 0.01) effects were found for eccentric force production during isokinetic testing (Fig. 3b). Additionally, negligible impact of condition was observed on eccentric force production during either the first (g = 0.03) or second attempts (g = 0.07).

#### Isometric testing

A trend for a main effect of condition (*p* = 0.07, η_P_^2^ = 0.19) and a significant main effect for time (*p* < 0.001, η_P_^2^ = 0.65) were identified for isometric force production at 120 degrees of knee extension (Fig. 3c). However, a significant interaction effect was not identified (*p* = 0.49, η_P_^2^ = 0.03). Follow-up analysis showed that Attempt 2 was approximately 148 N less than Attempt 1 with both conditions combined. Effect size analysis of the impact of condition on peak force production showed a negligible effect in favor of the energy drink condition during attempt 1 (g = 0.11) and a small effect in favor of the energy drink condition during the second attempt (g = 0.30).

No significant main (*p* > 0.17) or interaction (*p* = 0.89, η_P_^2^ = 0.00) effects were found for peak rate of force production during isometric testing at 120 degrees (Fig. 3e). However, effect size analysis of the impact of condition on peak rate of force production at 120 degrees showed a small effect in favor of the energy drink during attempt 1 (g = 0.23) and attempt 2 (g = 0.30). Similarly, no significant main (*p* > 0.28) or interaction (*p* > 0.16) effects were found for RFD_50_ or RFD_200_ at 120 degrees extension.

No significant main (*p* > 0.08) or interaction (*p* = 0.29, η_P_^2^ = 0.07) effects were found for maximal isometric force production at 150 degrees of knee extension (Fig. 3d). Effect size analysis of the impact of condition on peak force production showed a negligible effect in favor of the energy drink during attempt 1 (g = 0.13) and a negligible effect in favor of placebo during the second attempt (g = 0.05).

A main effect for time (*p* = 0.028, η_P_^2^ = 0.28) but not condition (*p* = 0.85, η_P_^2^ = 0.00) was found for peak rate of force production during isometric testing at 150 degrees (Fig. 3f). No significant interaction effect was identified (*p* = 0.24, η_P_^2^ = 0.09). Effect size analysis of the impact of condition on peak rate of force production at 150 degrees showed a negligible effect in favor of the energy drink condition during attempt 1 (g = 0.13) and a negligible effect in favor of placebo during attempt 2 (g = 0.05). Similarly, no significant main (*p* > 0.11) or interaction (*p* > 0.54) effects were found for RFD_50_ at 150 degrees extension. A main effect of time (*p* = 0.002, η_P_^2^ = 0.49) but not condition was found for RFD_200_ at 150 degrees extension, though no significant interaction was identified (*p* = 0.98, η_P_^2^ = 0.00). Follow up indicated that RFD_200_ was lower at the second attempt as compared to the first.

#### Leg press

**Fig. 4:**
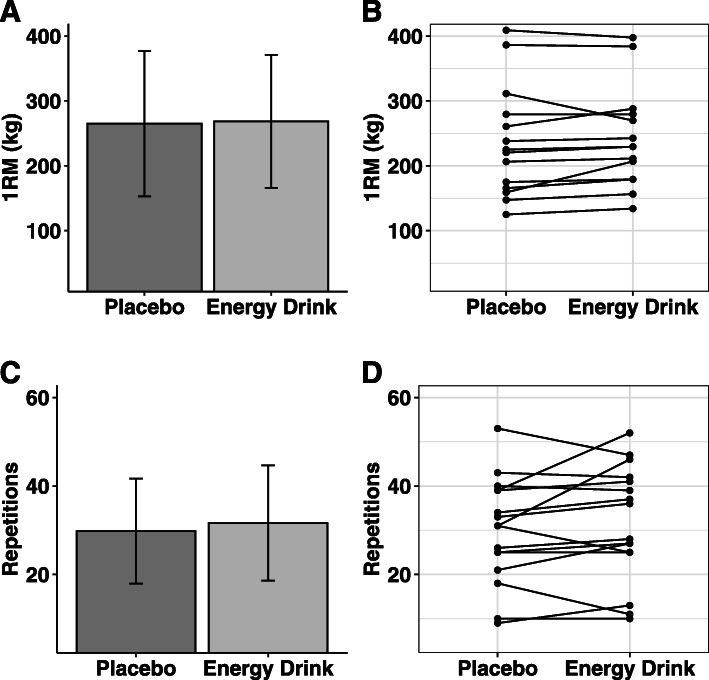
Leg Press Performance. Paired-samples t-tests were performed to examine group-level differences in leg press 1-repetition maximum (1RM; panel **a**) and repetitions to failure (panel **c**). No differences between conditions were observed. Error bars indicate SD, and individual responses are displayed in (panels **b** and **d**)

Significant differences in leg press one-repetition maximum (*p* = 0.46, g = 0.01) or repetitions to fatigue (*p* = 0.24, g = 0.06) were not identified between conditions (Fig. 4).

### Subjective measures

**Fig. 5:**
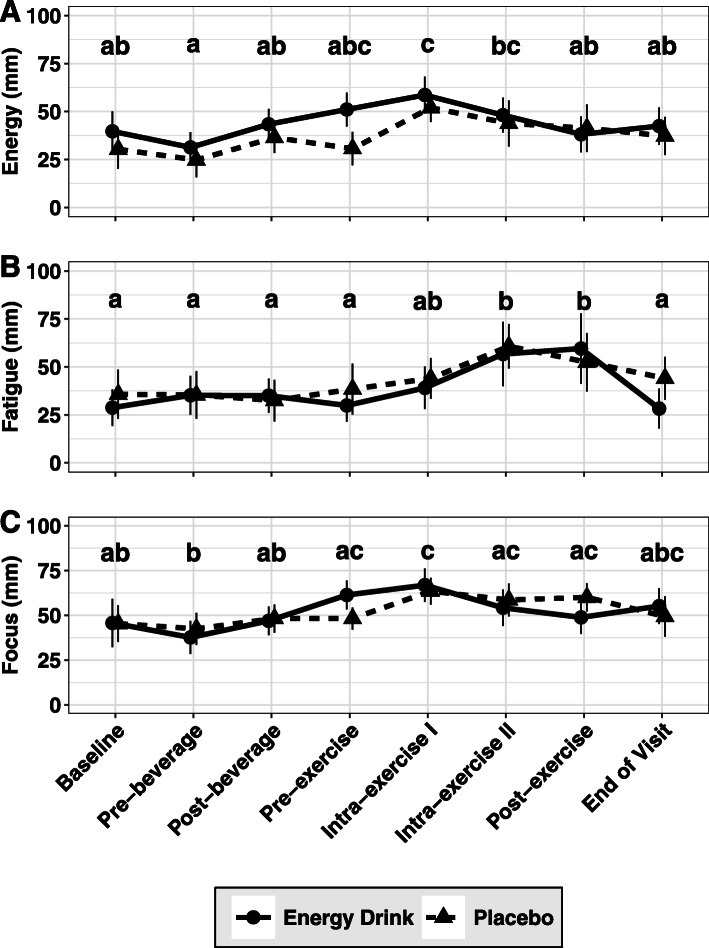
Subjective Variables. Subject evaluations of energy (**a**), fatigue (**b**) and focus (**c**), evaluated via digital analog scale, are displayed. Data were analyzed via ANOVA with repeated measures, and main effects of time were observed for all three variables. Results of Tukey post-hoc comparisons are displayed, with shared letters indicating no difference between time points. Error bars indicate the 95% confidence intervals for within-subjects SE due to the repeated-measures design of this study [24, 28]

Significant main effects of time were found for subjective measures of Energy (*p* = 0.008, η_P_^2^ = 0.26), Fatigue (*p* = 0.004, η_P_^2^ = 0.29), and Focus (*p* = 0.001, η_P_^2^ = 0.29). No significant main effects of condition (*p* > 0.086) or interaction effects (*p* > 0.010) were identified for any of the three subjective outcomes (Fig. 5).

## Discussion

The purpose of this investigation was to assess acute changes in force production, muscular endurance, and metabolism following consumption of a commercially available caffeine- and protein-containing energy drink. The results of this study suggest that in the context of a laboratory environment, consumption of the energy drink had minimal effect on lower body muscular performance measures, including maximal force production, muscular strength, endurance, and rate of force development. Similarly, consumption of the energy drink was not found to influence subjective ratings of energy, fatigue, or focus. However, the energy drink was found to significantly increase energy expenditure immediately following ingestion, as well as during the recovery period after a strenuous bout of exercise, as compared to a placebo containing negligible quantities of caffeine and protein. Finally, consumption of the energy drink had no demonstrable effect on RER, suggesting that changes in substrate utilization resulting from consumption were likely minimal.

In contrast to several previous studies, analysis of the performance data collected during this investigation revealed no significant between-condition differences for leg press one-repetition maximum, leg press repetitions to fatigue, eccentric or concentric force production during isokinetic testing, maximal isometric force production at 120 and 150 degrees of knee extension, and RFD characteristics at both 120 and 150 degrees of knee extension. Because caffeine has been shown to be the primary mediator of the acute ergogenic effects of energy drinks [4], the results of the present study may be explained by the amount of caffeine consumed by participants prior to performance testing or the specific performance outcomes examined. To date, the majority of investigations showing beneficial effects of caffeine consumption on measures of strength or power have employed doses of at least 5–6 mg·kg^− 1^ [29], with 6 mg·kg^− 1^ being the most common [30]. However, other research has indicated an unclear relationship between the dose of caffeine, ranging from 2 to 6 mg·kg^− 1^, and lower-body muscular strength and endurance [31]. Based on mean bodyweight, the male and female participants in the present study received acute doses of approximately 3.8 mg·kg^− 1^ and 4.4 mg·kg^− 1^, respectively. Interestingly, the null results of the present study align with an earlier investigation with similar performance outcomes conducted by our lab group [32]. Like the present investigation, no definitive improvements in isokinetic squat performance relative to placebo were identified when participants were provided a pre-workout supplement containing 300 mg (4.0 mg·kg^− 1^) and 225 mg (3.6 mg·kg^− 1^) of caffeine for males and females, respectively. In summary, it is possible that a greater acute dose of caffeine may be necessary for ergogenic effects to be detected using the performance outcomes employed in the present study or that caffeine has limited ergogenic value in the particular context of this study. While the participants in the present study were habitual caffeine consumers, it is unclear if this would influence the ergogenic effects of caffeine on resistance exercise [29]. Importantly, the notable differences between the laboratory and free-living settings should also be considered, particularly given the strong encouragement provided by researchers during all performance testing in the present study.

As hypothesized, EE was significantly increased immediately following ingestion of the caffeine and protein-containing energy drink, and remained elevated above baseline following the exercise bout. However, significant between-condition differences in RER were not detected, indicating that the proportions of carbohydrate and fat being oxidized were not measurably affected by consumption of the energy drink or placebo. However, the difference in protein content of the energy drink and placebo beverage, as well as the differential caffeine content, should be taken into consideration when interpreting these values. Importantly, EE in the energy drink condition was found to be approximately 0.77 kcal·min^− 1^ higher than placebo during the post-beverage time period, and approximately 0.37 kcal·min^− 1^ higher than placebo during the post-exercise time period. These results align with the findings of previous studies which reported significant elevations in EE following consumption of caffeine-containing energy drinks [11–13] as well as those which administered supplemental protein prior to exercise [15–17]. Several physiological mechanisms are responsible for these results. In addition to the well-documented thermogenic effects of caffeine [33, 34], it is highly likely that the whey and milk protein found in the product also contributed to the acute increases in metabolic rate observed by the present investigation. Because protein requires more energy to digest, absorb, and utilize compared to carbohydrates or fats [35], postprandial dietary thermogenesis has been shown to be higher following consumption of protein-rich foods compared to lower-protein controls [36]. However, without caffeine and protein-matched placebo conditions, it is not possible to accurately estimate the relative thermogenic contributions of each component of the energy drink used in the present study. Similarly, because the energy drink and placebo conditions utilized in the present study differed in energy content by ~ 100 kcal, some degree of the between-condition differences in EE should be attributed to the additional caloric content of the energy drink per se [36]. This limitation of the present investigation prevents the direct evaluation of different ingredients and macronutrient profiles on acute metabolic outcomes. As such, the practical applications of the present investigation pertain primarily to consuming vs. not consuming a caffeine- and protein-containing beverage for energy expenditure and resistance exercise performance outcomes. This could be relevant to those intentionally training with low carbohydrate availability and considering whether to exercise in a fasted state as compared to after an ingestion of a caffeinated, low-carbohydrate, high-protein beverage.

## Conclusions

The present investigation demonstrated that the acute consumption of a caffeine and protein-containing energy drink resulted in significantly increased resting and post-exercise energy expenditure compared to placebo, though some degree of this effect should be attributed to caloric differences between conditions per se. The energy drink was not found to influence respiratory exchange ratio or participants’ subjective ratings of fatigue, energy, and focus. Similarly, the energy drink exerted minimal effects on maximal force production, muscular endurance, and rate of force development compared to placebo within the context of this study. These results suggest that active individuals may improve acute metabolic outcomes both before and after exercise via consumption of a caffeine- and protein-containing energy drink. Additional information is needed regarding the effects of similar interventions on upper-body muscular performance as well as measures of sport-specific performance. Future investigations could include energy-matched conditions as well as protein and caffeine-matched placebo conditions to determine the direct contribution of caffeine and macronutrient composition on acute metabolic outcomes.

## Data Availability

The datasets used during the current study are available from the corresponding author upon reasonable request.

## Abbreviations

1RM: One-repetition maximum
ANOVA: Analysis of variance
ASA24®: Automated Self-Administered 24-Hour Dietary Assessment Tool
CV: Coefficient of variation
EE: Energy expenditure
RER: Respiratory exchange ratio
VAS: Visual analog scale
