# Correction to: Effects of Bang® Keto Coffee Energy Drink on Metabolism and Exercise Performance in Resistance-Trained Adults: A Randomized, Double-blind, Placebo-controlled, Crossover Study

**DOI:** 10.1186/s12970-020-00378-1

**Published:** 2020-09-15

**Authors:** Patrick S. Harty, Matthew T. Stratton, Guillermo Escalante, Christian Rodriguez, Jacob R. Dellinger, Abegale D. Williams, Sarah J. White, Robert W. Smith, Baylor A. Johnson, Mark B. Sanders, Grant M. Tinsley

**Affiliations:** 1 Energy Balance & Body Composition Laboratory, Department of Kinesiology & Sport Management, Texas Tech University, Lubbock, TX 79424 USA; 2 California State University, San Bernardino, California USA

**Correction to: J Int Soc Sports Nutr 17, 45 (2020)**

**https://doi.org/10.1186/s12970-020-00374-5**

Immediately after publication of this article [1], the authors noticed that in-text energy expenditure values had mistakenly been transferred from the incorrect table in the statistical output. This error occurred during revision of the article while addressing reviewer comments. These incorrect values occurred in the Abstract, Results and Discussions sections. However, the energy expenditure values presented in Fig. 2 are correct, and the accompanying statistical analysis was performed using the appropriate data.

The correct values in these sections are highlighted in bold below:

**‘Abstract’ section on page 2**

Following consumption of the beverage, EE was **0.18** kcal·min^-1^ greater than placebo at the post-beverage time point (*p* < 0.001) and **0.08** kcal·min^-1^ greater than placebo at the post-exercise time point (*p* = 0.011).

**‘Energy expenditure’ in ‘Results’ section on page 6**

However, EE increased by approximately **0.07** kcal·min^-1^ from post-beverage to post-exercise (*p* = 0.025). Additionally, post-exercise EE was approximately **0.13** kcal·min^-1^ higher than the baseline (pre-beverage) EE (*p* < 0.001).

Following consumption of the energy drink, EE increased approximately **0.24** kcal·min^-1^ from pre-beverage to post-beverage (*p* < 0.001) and remained elevated by approximately **0.21** kcal·min^-1^ at post-exercise (*p* < 0.001).

EE following consumption of the energy drink was **0.18** kcal·min^-1^ greater than placebo at the post-beverage time point (*p* < 0.001) and **0.08** kcal·min^-1^ greater than placebo at the post-exercise time point (*p* = 0.011).

**‘Discussion’ section on page 11**

Importantly, EE in the energy drink condition was found to be approximately **0.18** kcal·min^-1^ higher than placebo during the post-beverage time period, and approximately **0.08** kcal·min^-1^ higher than placebo during the post-exercise time period.

The authors apologize for the inconvenience caused.
